# Cellular response to bacterial infection in the grasshopper *Oxya chinensis*

**DOI:** 10.1242/bio.045864

**Published:** 2019-10-15

**Authors:** Xiaomin Zhang, Keshi Zhang

**Affiliations:** College of Life Science, Shanxi University, Taiyuan, Shanxi 030006, China

**Keywords:** Haemocyte, Histochemistry, Phagocytosis, Morphology, Wright-Giemsa staining

## Abstract

*Oxya chinensis* is one of the most widespread grasshopper species found in China and one of the most common pests against rice. In view of the importance of haemocytes in insect immunity in general, and the lack of information on the haemocytes of *O. chinensis*, we examined the haemocytes of this species in detail. We challenged the cellular response of this grasshopper with the bacteria *Escherichia coli*, *Staphylococcus aureus*, and *Bacillus subtilis*. Haemocyte morphology was observed using light, scanning electron and transmission electron microscopy, which revealed distinct morphological varieties of haemocytes. Granulocytes and plasmatocytes responded to the bacterial challenge by phagocytosis. Histochemical staining indicated the presence of acid phosphatase in plasmatocytes and granulocytes. We also observed non-phagocytic prohemocytes and vermicytes, but their functions in the circulation are unclear. Insect haemocytes play a crucial role in cellular immunity, and further research is needed for a comprehensive understanding.

## Abstract (precis)

**Summary:** We examined the cellular response of *Oxya chinensis* against bacterial challenges. We showed that two types of haemocytes shared the duty of phagocytosis.

## INTRODUCTION

Insect haemocytes have drawn scientists' attention since they were first discovered by Swammerdam (1637–1680) (Jones, 1962), and interest in them has grown with knowledge of their crucial role in the insect immunity. Insects respond to pathogenic challenges through an interplay of two mechanisms, humoral and cellular defences (Charles and Killian, 2015; Urbański et al., 2018). Haemocytes mediate cellular defence by carrying out roles such as phagocytosis, nodulation and encapsulation, and also facilitate humoral defence by synthesising and releasing enzymes and other immune factors (Charles and Killian, 2015; Hillyer, 2016; Urbański et al., 2018).

Insect haemocytes comprise distinct populations, which vary in their morphology and function (Strand, 2008). The origin, function, and classification of these haemocytes are debatable and not fully understood (Duressa and Huybrechts, 2016), partly due to lack of a standardised protocol for insect immune investigation. Direct comparison of the insect immune response is difficult due to the variety of challenges used, life stages examined and methodologies practised (Charles and Killian, 2015). The nomenclature of the haemocytes is not normalised across Insecta (Hillyer, 2016), but recent studies have attempted to generalise variations. Several subtypes have been recognised (Castillo et al., 2006; Grigorian and Hartenstein, 2013; Hillyer and Strand, 2014; King and Hillyer, 2013; Ribeiro and Brehélin, 2006). The most often observed include prohemocytes, which are hypothetical, small, spherical haemocyte progenitors with a high nucleus-to-cytoplasm ratio. Plasmatocytes are spindle-shaped cells with cytoplasmic projections and an absence of granules in the cytoplasm. They function in phagocytosis and encapsulation. Granulocytes (or granular cells) are spheroid and oval cells, which contain uniformly sized and electron-dense cytoplasmic granules, and act as phagocytes. Oenocytoids are large, uniformly shaped cells with a low nucleus-to-cytoplasm ratio and an eccentrically located nucleus. They contain phenoloxidase, and are responsible for haemolymph darkening (melanisation). Finally, spherulocytes, which may be a source of cuticular components, are irregularly shaped cells packed with large inclusions (spherules). Rarer insect haemocytes, which are only occasionally observed, include cystocytes, adipohemocytes, vermicytes and megakaryocytes (Akai and Sato, 1979; Brehélin et al., 1978; Gillespie et al., 2000; Gupta, 1979; Jones, 1977; Ren et al., 2014; Ribeiro and Brehélin, 2006). Cystocytes (or coagulocytes) are oval or fusiform cells usually lysed or degranulated *in vitro*, which differ from granulocytes under the periodic acid-Schiff reaction. Adipohemocytes are spherical or oval cells, with the presence of reflective fat droplets, non-lipid granules and vacuoles. Vermicytes (podocytes or vermiform cells) are irregularly shaped cells with multiple cytoplasmic extensions and small electron-dense granules. Megakaryocytes are large cells filled by nuclei, with minimal cytoplasm. Knowledge of the functions of these rarer haemocyte types is limited.

Grasshoppers play an essential role in the grassland ecosystem and have a considerable economic impact on agriculture (Duressa et al., 2015; Latchininsky, 2013). *O. chinensis* is the most widely distributed grasshopper species in China, and one of the major pests of rice, maize, sorghum and wheat (Liu et al., 1999; Zhang and Huang, 2008). Insect haemocytes have been studied extensively in *Drosophila melanogaster* and selected lepidopteran species (Lavine and Strand, 2002). However, previously published literature on *O. chinensis* haemocyte morphology is limited to our early study, Wang et al. (2011), in which we employed three stains to compare their efficiency. Here, we aimed to provide a more comprehensive understanding of the haemocytes of the grasshopper *O. chinensis*, and insights into standardising insect haemocyte examination methods. The cellular response of *O. chinensis* was elicited by injection with live *Escherichia coli*, *Bacillus subtilis* and *Staphylococcus aureus*. These bacteria are frequently used in insect immune studies due to their non-pathogenicity for insects (Arp et al., 2017). Light microscopy was used with multiple stains to examine haemocyte morphological and histochemical characteristics, and the cellular response to bacterial challenge. Scanning and transmission electron microscopy were used to reveal additional details of the haemocytes.

## RESULTS

### Haemocyte morphology

**Fig. 1.**
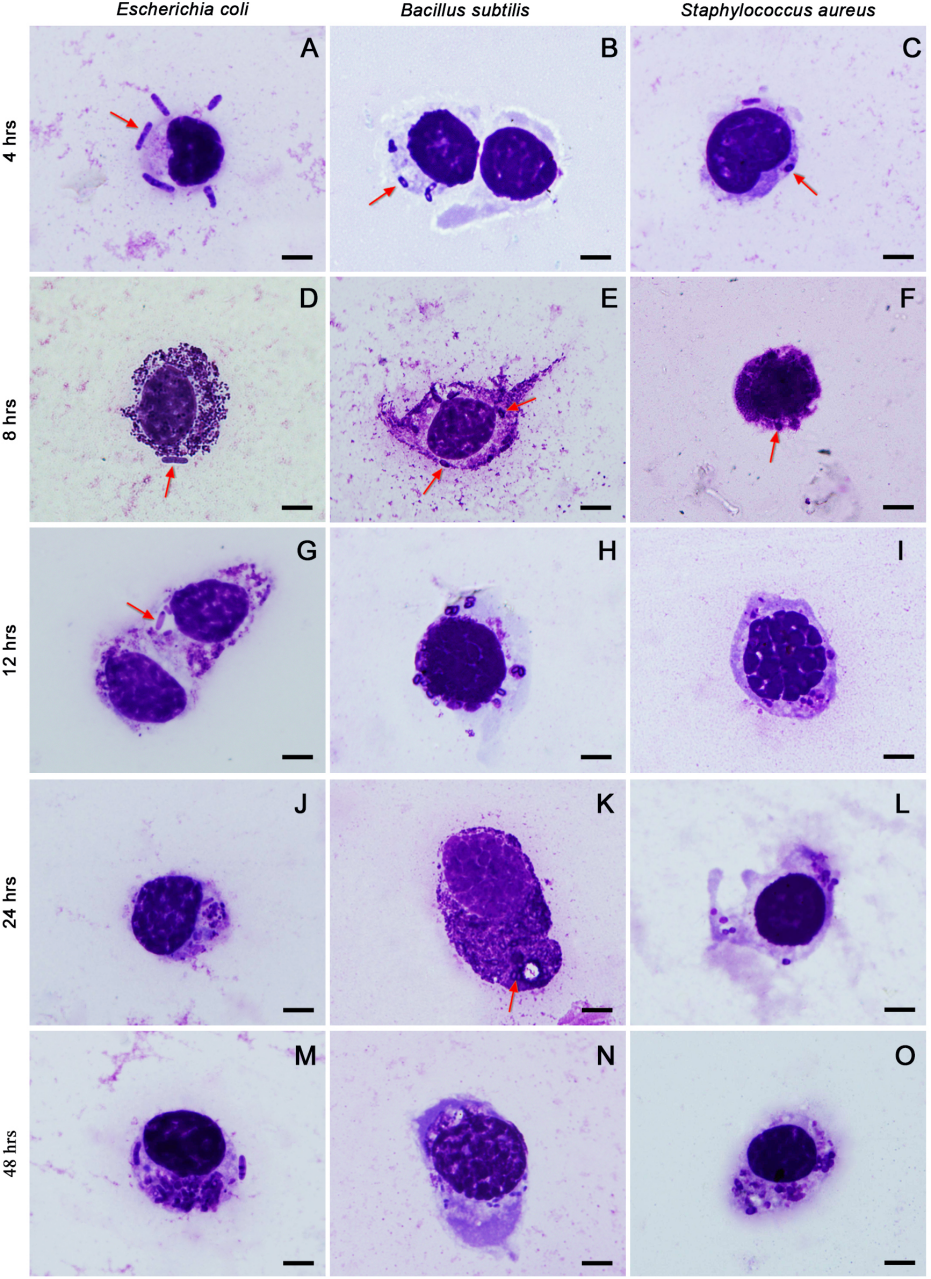
**The phagocytic response of the grasshopper *O. chinensis* against *E. coli*, *B. subtilis* and *S. aureus* bacteria at 4, 8, 12, 24 and 48 h post-injection.** The phagocytes shown are plasmatocytes (A–C,H–J,L–O) and granulocytes (D–G,K). Arrows point to bacteria. Scale bars: 5 μm.

The haemocytes from *O. chinensis* varied regarding their shape, size and cytoplasmic contents. They responded to the bacterial challenges by phagocytosis (Fig. 1). The bacteria *E. coli*, *B. subtilis* and *S. aureus* were bound and digested internally by phagocytes. The phagocytes were capable of digesting the engulfed bacteria while attaching new ones. Between 4 and 8 h after injection, most bacteria were attached to the haemocyte membrane, while some were being ingested. After 12 h, most bacteria inside the phagocytes were broken down into fractions. However, binding of bacteria by phagocytes was still observed at the end of the experiment. Thus, complete removal of the injected bacteria by *Oxya*’s immune system at the given dose requires more than 2 days. The bacteria *B. subtilis* formed endospores, as shown by their transparent centre, and were digested by the *O. chinensis* phagocytes. The unstained centre was not seen in *E. coli* or *B. subtilis*.

**Fig. 2.**
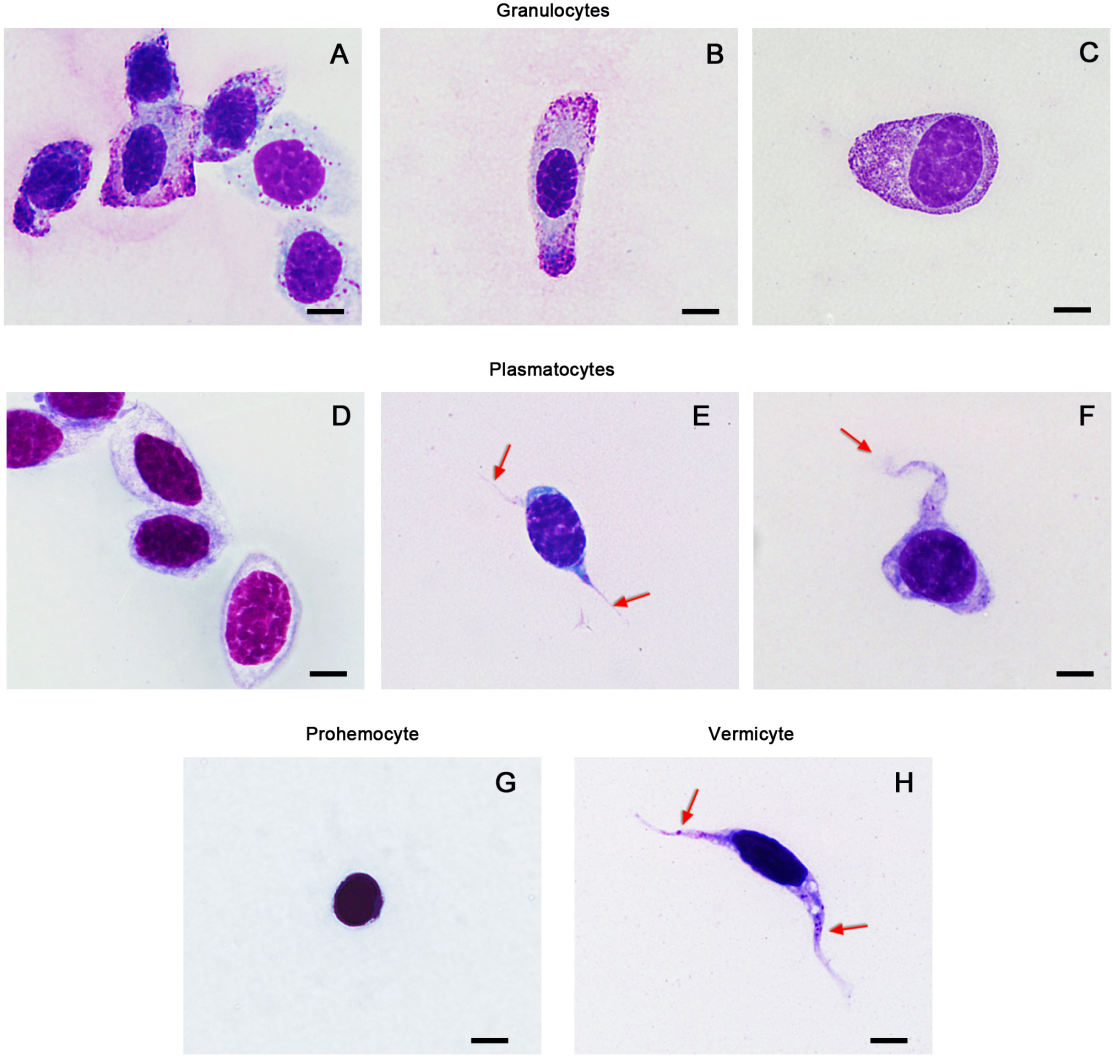
**Wright-Giemsa-stained haemocytes of *O. chinensis* under the light microscope.** (A–C) Polymorphic granulocytes with purple cytoplasmic granules, which vary in density. (D) Oval-shaped plasmatocytes. (E,F) Plasmatocytes with extended pseudopodia (arrows). (G) A spheroid prohemocyte with limited cytoplasm. (H) A worm-shaped vermicyte with elongated cytoplasm and a nucleus containing some cytoplasmic granules (arrows). Scale bars: 5 μm.

The phagocytes of *O. chinensis* contained two morphological varieties, which were distinguished by the presence of cytoplasmic granules. Round, oval and irregularly shaped granulocytes contained small (<1 µm) basophilic granules (purple), and measured 12–34 µm in diameter (Fig. 2). The centrally located nucleus was stained dark red or bluish purple with Wright-Giemsa and measured 7–20 µm in diameter. The cytoplasm of the granulocytes was stained transparent (neutral), pink (eosinophilic) or light blue (basophilic). The density of the granules ranged from sparse to packed, and their distribution was uniform or erratic.

**Fig. 3.**
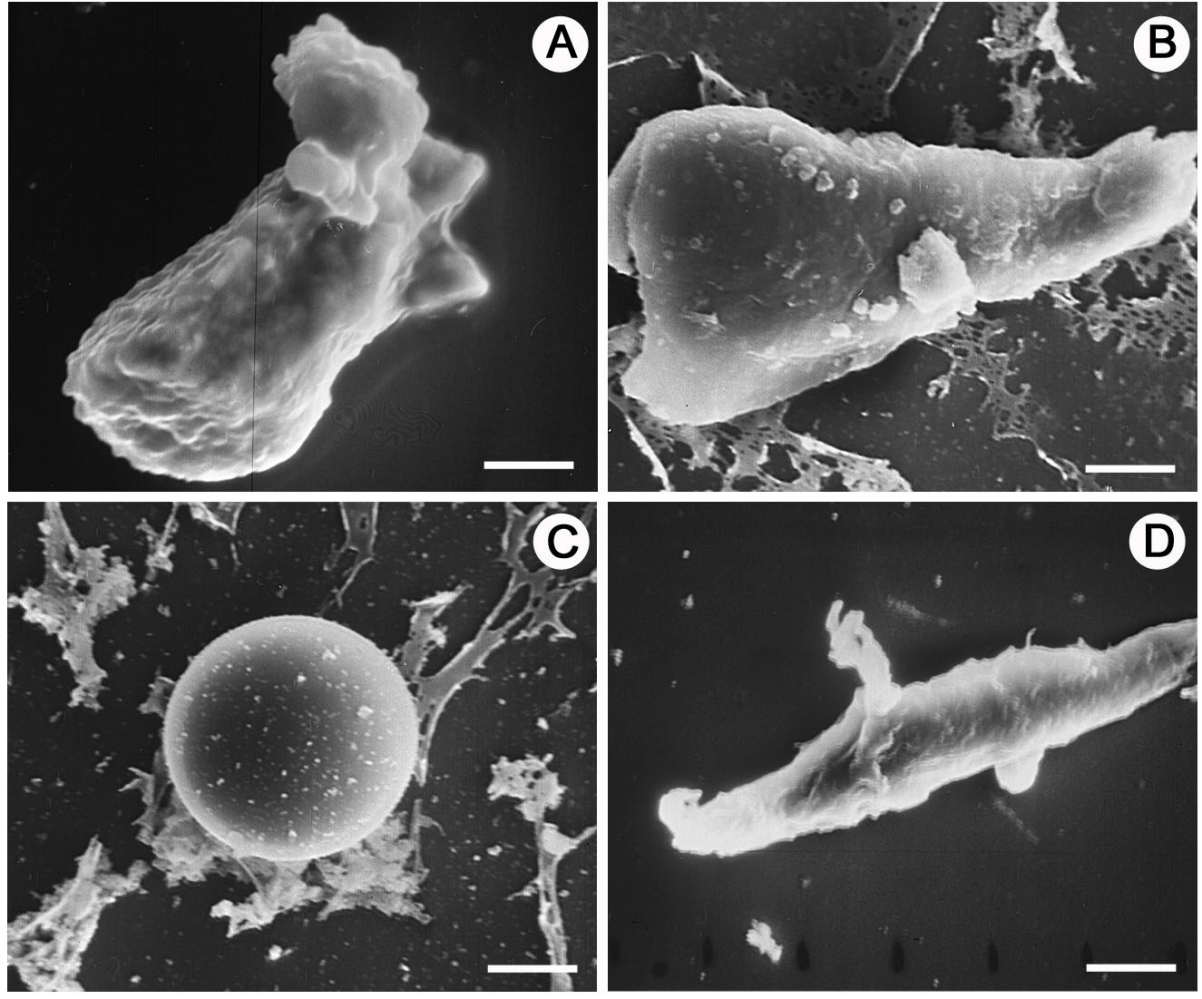
**Haemocytes of *O. chinensis* under the scanning electron microscope.** (A) An irregularly shaped granulocyte with lumps on the membrane caused the presence of cytoplasmic granules. (B) An irregularly shaped plasmatocyte with a relatively smooth membrane. (C) A spheroid prohemocyte with smooth membrane. (D) A vermicyte with rough membrane and pseudopodia. Scale bars: 7 μm (A,D), 5 μm (B), 4 μm (D) .

**Fig. 4.**
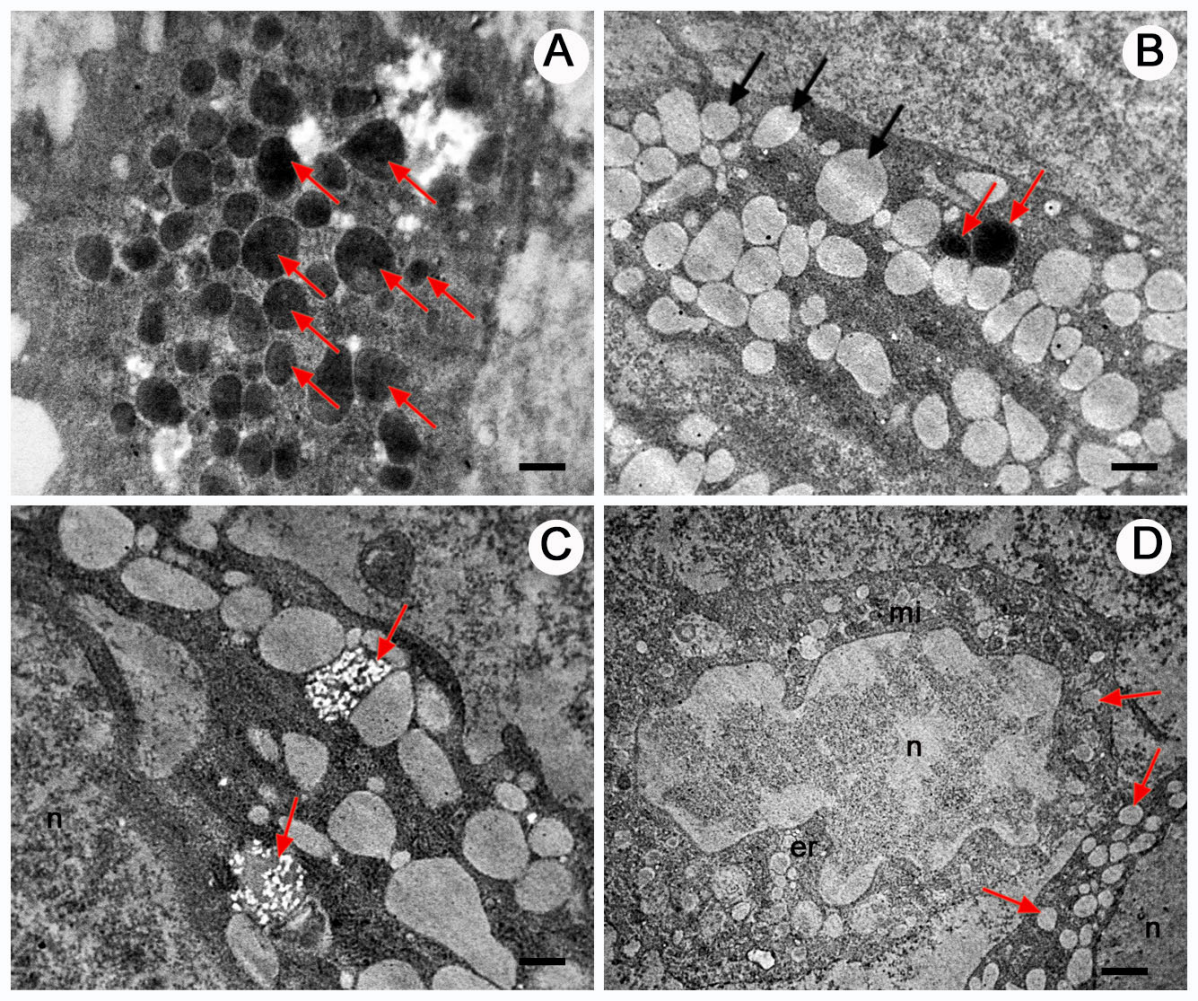
**Granulocytes of *O. chinensis* under the transmission electron microscope.** The slides depict sections of granulocytes with visible granules, indicated by arrows. (A,B) Electron-dense (red arrows) and electron-lucent (black arrows) granules in the cytoplasm are shown. (C) Section of a granulocyte with two clusters of small bright inclusions (arrows). (D) Arrows indicate electron-lucent granules. The nucleus (n), mitochondria (mi) and endoplasmic reticulum (er) are found in the cytoplasm. Scale bars: 0.5 μm (A), 0.8 μm (B), 0.6 μm (C), 1 μm (D).

Under the scanning electron microscope, granulocytes showed a rough surface (Fig. 3). The small lumps on the cell membrane are likely cytoplasmic granules, and we used the presence of these spherical swellings to distinguish granulocytes from the other haemocytes. Under the transmission electron microscope, the electron-dense and electron-lucent granules in granulocytes were round or oval, and without regular structure (Fig. 4). In addition to the granules, mitochondria, endoplasmic reticulum, phagosome-like vacuole and clumps of small bright inclusions were also visible (Fig. 4D).

**Fig. 5.**
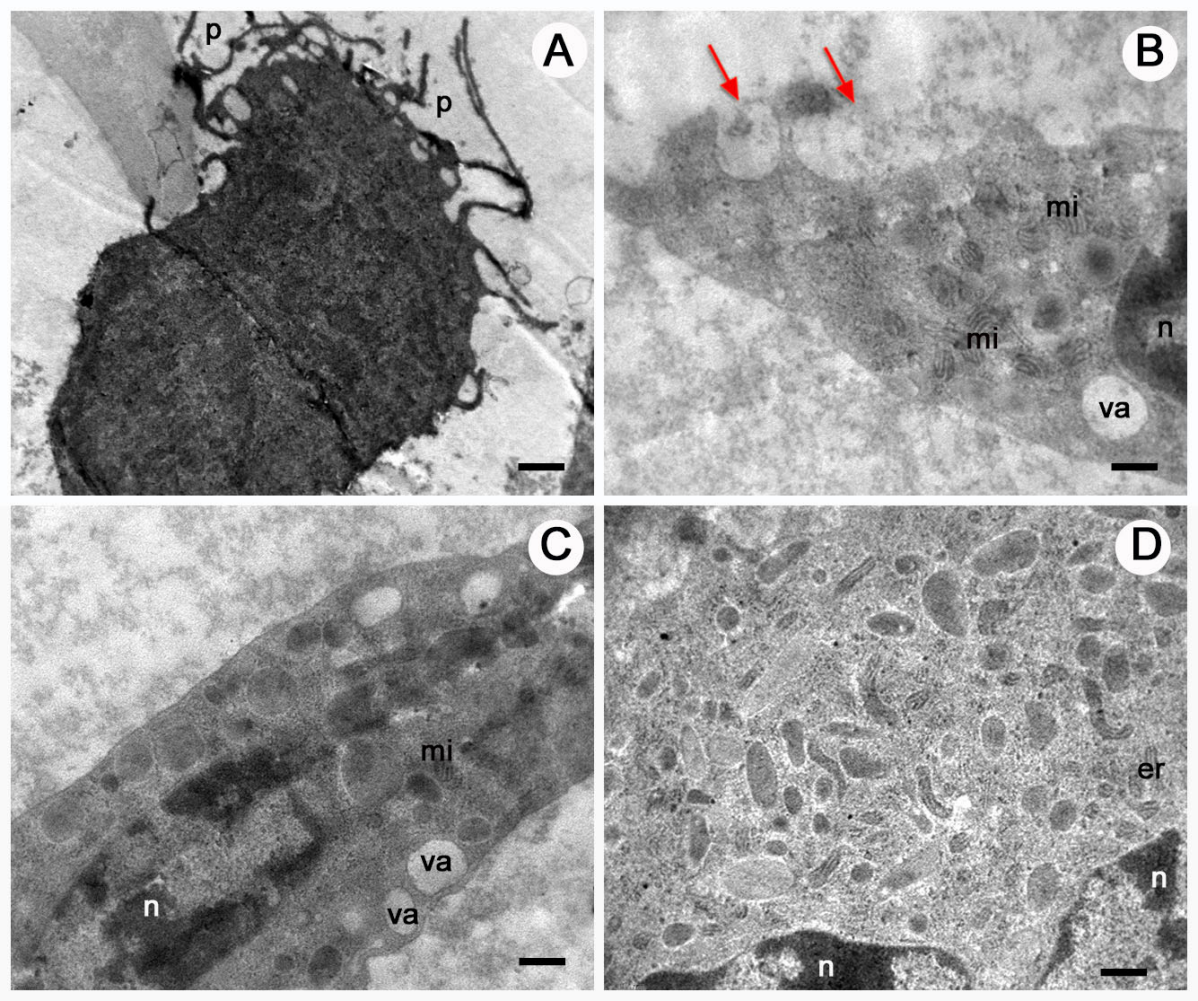
**Plasmatocytes of *O. chinensis* under the transmission electron microscope.** (A) Many elongated pseudopodia (p) extended outward from the plasmatocytes. (B) Arrows indicate exocytosis-like activity. (C,D) Nuclei (n), mitochondria (mi), endoplasmic reticulum (er), Golgi apparatus (go) and vacuole (va) were observed in the cytoplasm. Scale bars: 1 μm (A), 0.25 μm (B), 0.5 μm (C,D).

The phagocytes without granules (plasmatocytes), were polymorphic with a round or oval, irregular, spindle-like shape, and measured 10–32 μm in diameter (Fig. 2). Cytoplasmic projections including pseudopodia were seen under the light, scanning electron and transmission electron microscopes (Figs 3 and 5). The nuclei of plasmatocytes were stained blue or purplish-red with Wright-Giemsa and measured 8–18 μm in diameter. Plasmatocyte cytoplasm was uniformly distributed around the nucleus and stained blue, pinkish-red or transparent. Under the scanning electron microscope, the outer surface of plasmatocyte cell membranes was relatively smooth. Under the transmission electron microscope, mitochondria, endoplasmic reticulum, Golgi apparatus, vacuoles and other inclusions were recognised in the cytoplasm (Fig. 5). Exocytosis-like activities were observed (Fig. 5B).

**Fig. 6.**
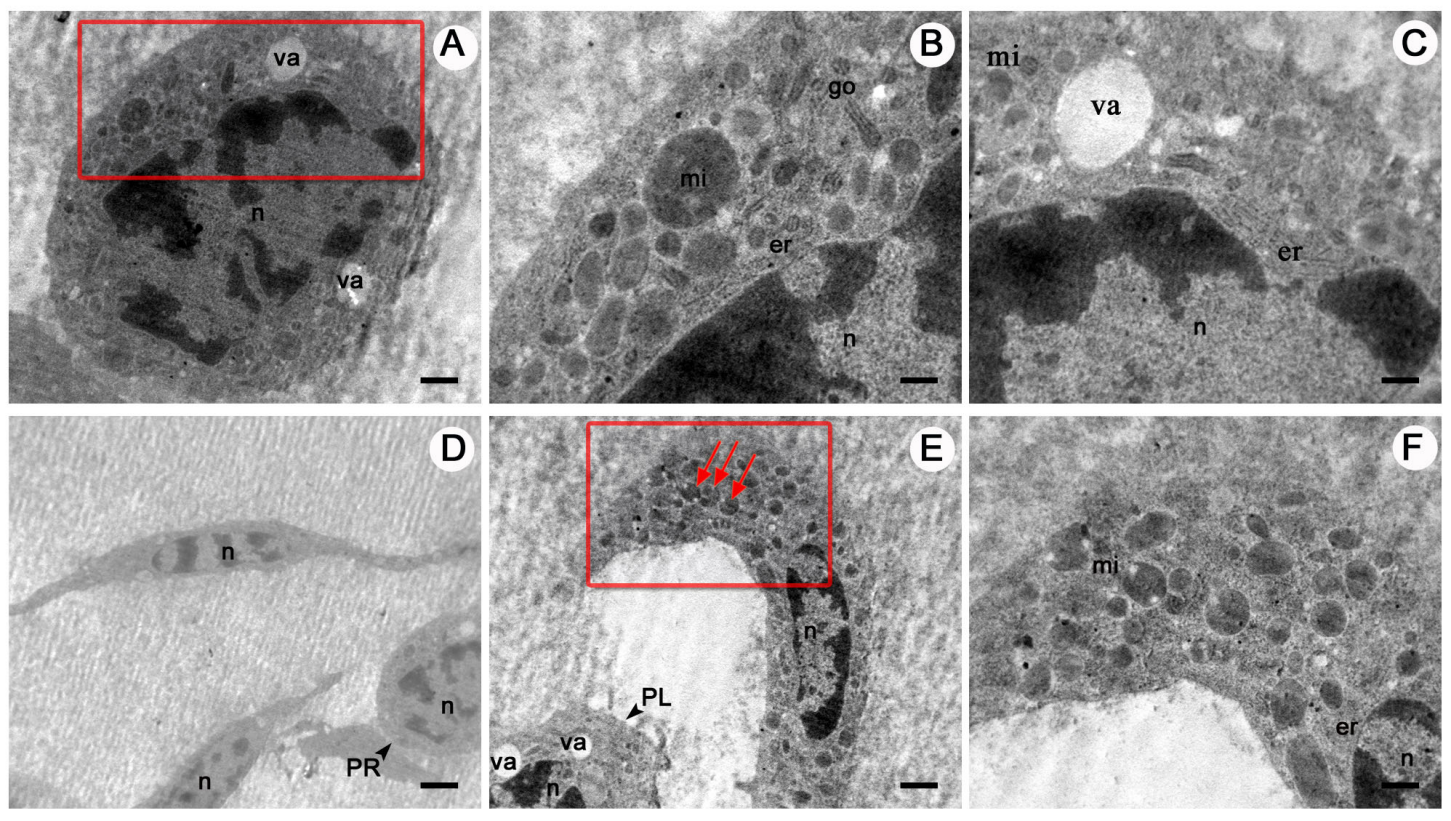
**A prohemocyte and vermicytes of *O. chinensis* under the transmission electron microscope.** (A–C) Prohemocytes (A). B and C are enlarged sections of A (red rectangle). Golgi apparatus (go), nuclei (n), mitochondria (mi), endoplasmic reticulum (er) and vacuole (va) are visible in the cytoplasm. (D–F) Vermicytes of *O. chinensis* under the transmission electron microscope. (D) Two worm-shaped vermicytes with elongated cytoplasm and nuclei. (E) Arrows indicate some of the vermicytes' cytoplasmic inclusions. (F) An enlarged section of E (red rectangle). Nuclei, mitochondria, endoplasmic reticulum, vacuole, and additional prohemocytes (PR) and plasmatocytes (PL) were also observed. Scale bars: 1 μm (A), 0.5 μm (B,C,E,F), 2.5 μm (D).

Two non-phagocytic varieties of haemocytes, prohemocytes and vermicytes, were sporadically observed in this study. These two cell types were morphologically distinct from plasmatocytes and granulocytes. Prohemocytes were much smaller than the other cell types, and displayed a spheroid shape approximately 8–12 µm in diameter (Fig. 2). The large nucleus was dyed purple or violet with Wright-Giemsa, which almost filled the whole cell. Prohemocytes had a smooth cell membrane under the scanning electron microscope with no pseudopodia or other cytoplasmic projections (Fig. 3). Under the transmission electron microscope, mitochondria, endoplasmic reticulum, Golgi apparatus, vacuoles and other electron-dense and electron-lucent particles were observed in the cytoplasm (Fig. 6).

Vermicytes, or vermin-formed haemocytes, were found to have distinctive worm-shaped cytoplasm and elongated nuclei (Fig. 2). The span of some vermicyte extensions was over 30 μm. Unlike plasmatocytes with elongated pseudopodia, vermicyte nuclei were attenuated and elongated with the cell. Stained dark blue by Wright-Giemsa, these nuclei were 8–25 μm in diameter. Granules were found in the cytoplasm of many vermicytes examined. Pseudopodia were noticed under the scanning electron microscope (Fig. 3D). Under the transmission electron microscope, vermicyte cytoplasm was abundant in the endoplasmic reticulum, mitochondria and other inclusions (Fig. 6).

### Haemocyte histochemistry

**Fig. 7.**
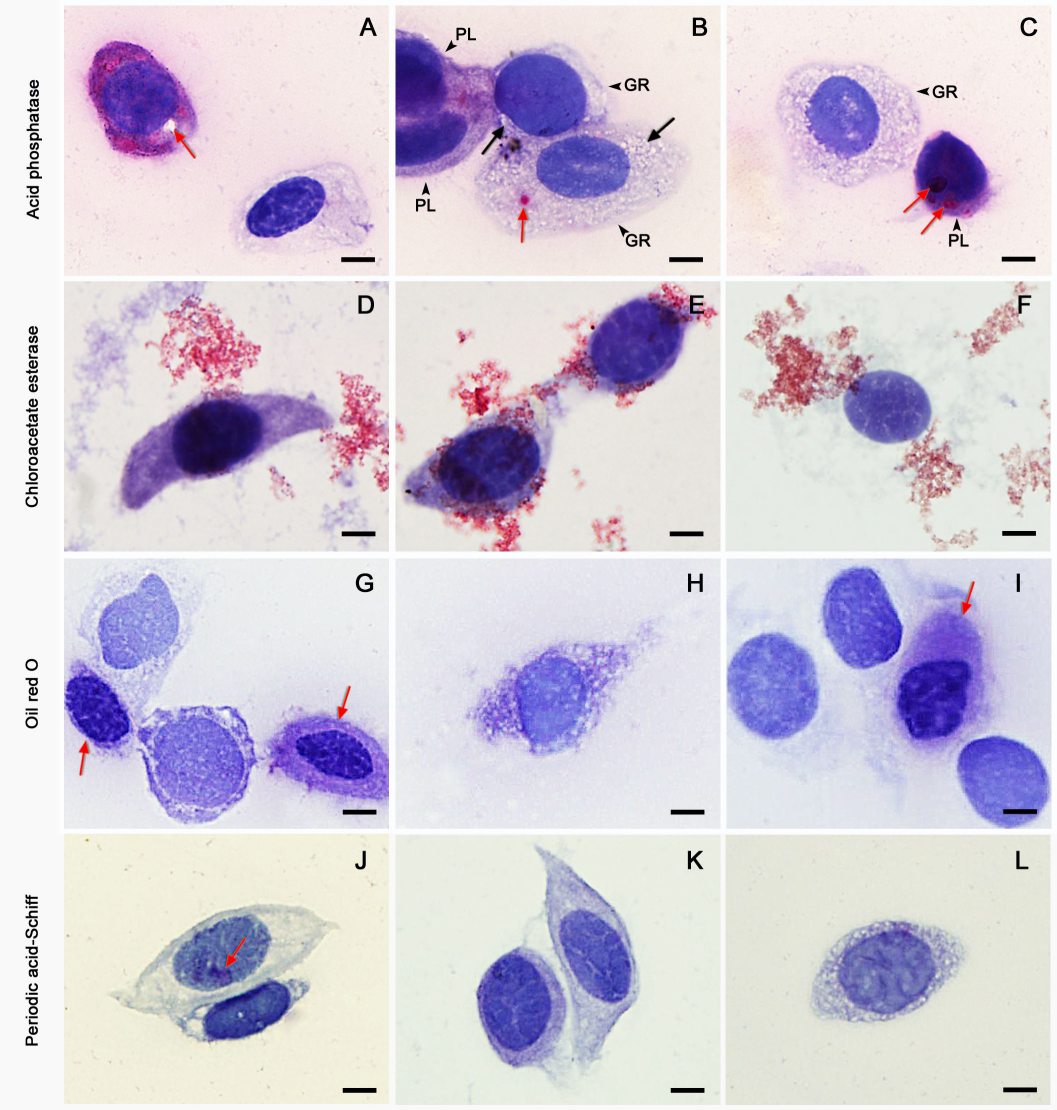
**Histochemically stained haemocytes of *O. chineneis*.** (A–C) Purplish-red colouration indicates a positive reaction in haemocytes stained with ACP. (A) Two ACP-positive plasmatocytes, one of which contains an unstained vacuole (arrow). (B) Two weakly ACP-positive plasmatocytes (black arrows) and two granulocytes with one containing an ACP-positive red granule (red arrow). (C) An ACP-positive plasmatocyte and an ACP-negative granulocyte. The two red arrows point to different sized ACP-positive red granules. (D–F) Haemocytes stained with CAE reaction. The haemocytes are CAE-negative, while the background is positively reacted and red in colour. Plasmatocytes are shown in D and E. (F) A naked nucleus with no conspicuous cytoplasm. (G–I) Haemocytes stained with ORO. (G) Two weakly ORO-positive plasmatocytes (red arrows). (H,L) Granulocyte with clear granules, indicating negative ORO and PAS reactions. (I) A weakly ORO-positive plasmatocyte (arrow) and three nuclei with no defined cytoplasm. (J–L) Haemocytes stained in PAS reaction. (J) One of the two plasmatocytes has PAS-positive purplish-red substances (arrow). (K) Two negatively reacted plasmatocytes. Scale bars: 5 μm.

Plasmatocytes and granulocytes were found to contain acid phosphatase (ACP), indicated by a purplish-red colouration of the cytoplasm (Fig. 7). ACP-negative plasmatocytes and granulocytes were also observed with cytoplasm that was clear or filled with clear refractive granules. Chloroacetate Esterase (CAE) staining indicated the presence of a positively reacted compound in the haemolymph and outside the haemocytes. No positively reacted lipid droplets were revealed by Oil Red O (ORO) staining inside haemocytes. The ORO-stained cytoplasm of some haemocytes was distinctively darker than others. A few haemocytes showed a positively reacted pinkish-red substance with periodic periodic acid-Schiff (PAS) staining.

### Differential haemocyte count

**Fig. 8.**
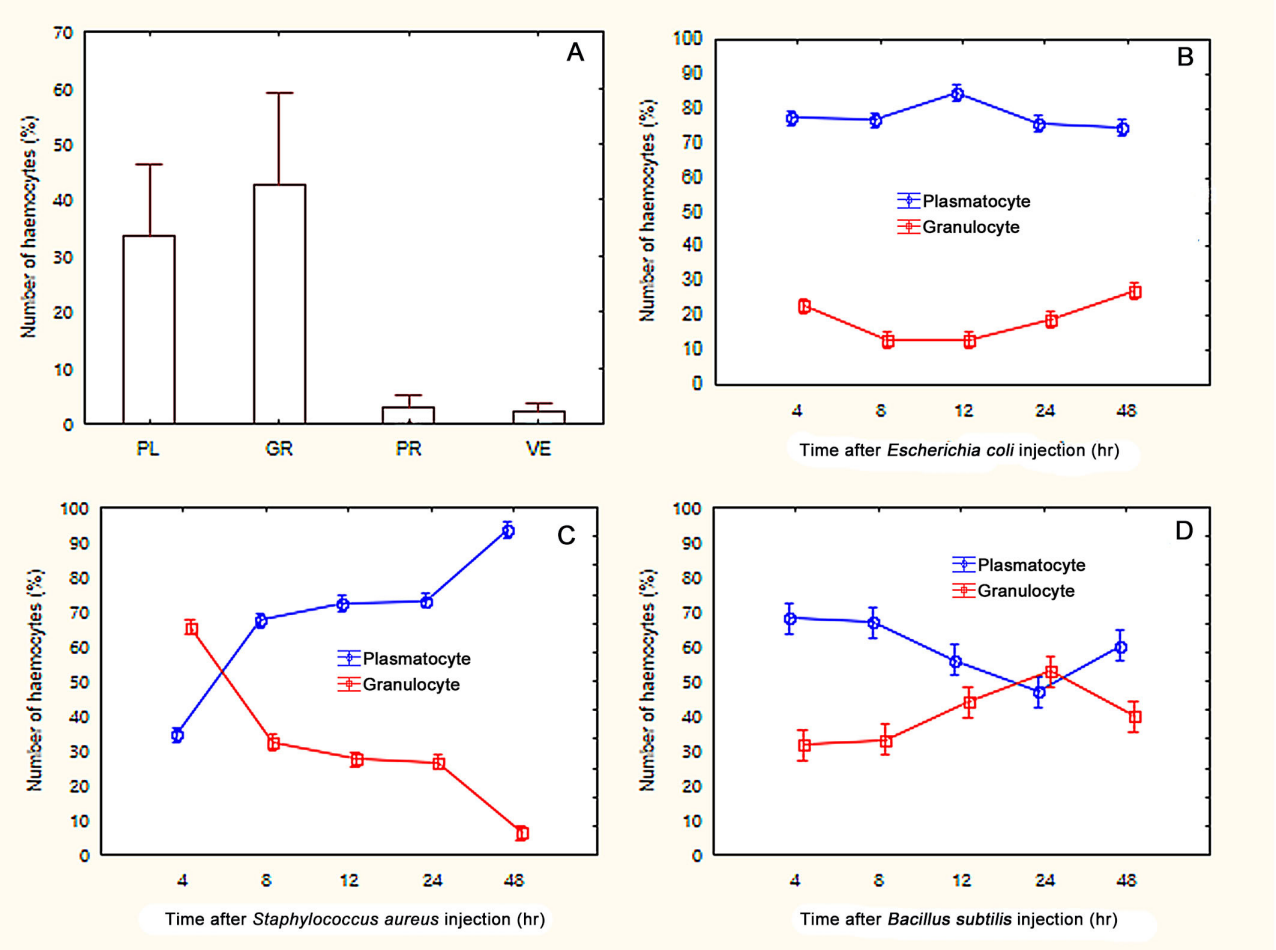
Haemocyte counts from the Wright-Giemsa-stained preparations of normal adults (A), and phagocyte proportion of adults injected with (B) *Bacillus subtilis*, (C) *Escherichia coli* and (D) *Staphylococcus aureus*. (A) Prohemocyte (PR), plasmatocyte (PL), granulocyte (GR) and vermicyte (VE). (B-D) The percentages of plasmatocytes and granulocytes with and without attachment or engulfment of injected bacteria are shown on the y-axes.

Plasmatocytes and granulocytes were the most common haemocytes found in this study, and comprised approximately 90% of the total cells counted (Fig. 8). Slightly more granulocytes were found than plasmatocytes in the untreated grasshopper samples. However, in the bacteria-treated samples, significantly more plasmatocytes were found than granulocytes, especially with grasshoppers injected with *E. coli* and *S. aureus*. The changes in amounts of plasmatocytes and granulocytes were inconsistent across the three bacteria-treated groups. The number of plasmatocytes and granulocytes were relatively uniform in the *E. coli-*treated group. The number of plasmatocytes and granulocytes in individuals treated with *B. subtilis* and *S. aureus* varied during the 48-h period. The number of plasmatocytes increased, while granulocytes decreased, in individuals treated with *S. aureus*. In contrast, granulocytes increased, but plasmatocytes decreased, in the *S. aureus*-treated group.

## DISCUSSION

Haemocytes of *O. chinensis* can be classified functionally as phagocytes and non-phagocytic haemocytes, or morphologically as granulocytes, plasmatocytes, prohemocytes and vermicytes. Phagocytes, or more specifically, granulocytes and plasmatocytes, account for the majority of haemocytes observed in this study. Phagocytosis is the primary defensive strategy utilised by insects against bacterial infection, and as a result, phagocytes account for a substantial proportion of insect haemocytes (Blandin and Levashina, 2007; Hillyer, 2016). The capacity of *Oxya*’s plasmatocytes and granulocytes for digesting endocytosed materials was indicated by our observations with hydrolase and acid phosphatase. Hydrolase, located in the lysosomes of haemocytes, is responsible for breaking down endogenous and exogenous macromolecules, including the bacterial cell wall (Callewaert and Michiels, 2010; Wu and Yi, 2015). Additionally, insect haemocytes release acid phosphatase into the haemolymph to modify the pathogens' surface molecular structure, thus enhancing the phagocytotic response (Callewaert and Michiels, 2010).

The phagocytes of *O. chinensis* are morphologically and functionally similar to those of most other insects. High abundance of plasmatocytes and granulocytes has also been observed in other grasshoppers and locusts (Anggraeni et al., 2011; Yu et al., 2016). Conversely, the structured granules of granulocytes previously observed by transmission electron microscopy (Ribeiro and Brehélin, 2006) were not seen in this study. Granulocytes have mostly been reported to be smaller than plasmatocytes, at 5–9 μm (Hillyer and Strand, 2014; Ribeiro and Brehélin, 2006), but we found them to be similarly sized to plasmatocytes in *O. chinensis*. In Lepidoptera, plasmatocytes are mostly seen in the role of encapsulation rather than phagocytosis (Ribeiro and Brehélin, 2006).

Granulocytes of *Locusta migratoria* were found to contain lysozyme in their granules (Zachary and Hoffmann, 1984). However, we observed that the granules of granulocytes did not elicit phagocytosis by haemocytes. In *O. chinensis*, both plasmatocytes and granulocytes participated in phagocytosis of injected bacteria.

Our findings agree with previous observations that not all plasmatocytes or granulocytes are involved in direct phagocytosis (Yu et al., 2016). Some *O. chinensis* plasmatocytes and granulocytes contained no acid phosphatase and did not respond to bacterial intruders. Granulocytes have been shown to participate in the activation of the encapsulation response, which releases opsonin-like materials in the presence of foreign objects (Browne et al., 2013; Hillyer, 2016). These materials cause the deformation of plasmatocytes to assist formation of capsules consisting of multiple layers around the unwanted large substances (Ribeiro and Brehélin, 2006). Other functions of granulocytes, such as haemolymph clotting, wound healing and melanisation were reported in the greater wax moth *Galleria mellonella*, the migratory locust *L. migratoria* and mosquitoes (Akai and Sato, 1979; Dushay, 2009; Hillyer and Strand, 2014).

Vermicytes are distinguished from plasmatocytes and granulocytes based on their morphology, histochemistry, and function. The small inclusions of vermicytes and their non-phagocytic nature have been used to separate them from the other haemocytes (Ribeiro and Brehélin, 2006). Most studies have considered vermicytes as a sub-class or variant form of plasmatocytes, and they are rarely reported in insects (Gupta, 1979; Jones, 1977; Ribeiro and Brehélin, 2006).

None of the *O. chinensis* haemocytes we observed morphologically corresponded to adipohemocytes, oenocytoids, podocytes or megakaryocytes. Our ORO experiment found no lipid droplets in the haemocytes of *O. chinensis*, indicating an absence of adipohemocytes. ORO reveals neutral lipids such as triglycerides and cholesterol esters (Tame et al., 2018). Under the transmission electron microscope, the lipid droplets measuring about 1 μm in diameter that have been observed to fill adipohemocytes from the Chinese grasshopper *Acrida cinerea* (Yu et al., 1977), were not observed in *O. chinensis*.

In this study, cystocytes or coagulocytes were not considered a distinct haemocyte type in *O. chinensis*. Our results are in contrast to those of Costin (1975) in separating cystocytes from plasmatocytes and granulocytes histochemically with the periodic acid-Schiff stain. Structured granules or globules were seen by transmission electron microscopy in a study of *Locusta migratoria* (Brehélin et al., 1978), but we did not see these in *O. chinensis*. Early studies described cystocytes or coagulocytes as having fewer granules than granulocytes, and interpreted the cytoplasmic remnants as coagulum or coagulated haemolymph (Costin, 1975; Gillespie et al., 2000; Gupta, 1979), while later studies have not mentioned their existence as a separate type. Cystocytes of *L. migratoria* have been classified as a subpopulation of granulocytes, which contain less red granules and participate in phagocytosis (Yu et al., 2016). The most reported role of cystocytes is the coagulation of insect haemolymph (Gupta, 1979; Zachary and Hoffmann, 1984). However, the coagulation process has been reported to require the cooperation of haemocytes and the plasma (Dushay, 2009). Granulocytes in many species have been suggested to function in coagulation (Grigorian and Hartenstein, 2013; Hillyer and Strand, 2014).

In *O. chinensis*, we found more plasmatocytes than granulocytes in bacteria-infected samples, which differs from results in *O. japonica*, in which more granulocytes were found (Anggraeni et al., 2011). However, this discrepancy may be due to differences in the bacterial challenges used by the two studies. We found that replenished or enhanced abundance of plasmatocytes and granulocytes were associated with the cellular response in *O. chinensis*. This has also been seen in a study of *L. migratoria*, in which increases in granulocytes and plasmatocytes after inoculation with the fungus *Metarhizium acridum* were suggested to result from a release of sessile haemocytes into the circulation (Yu et al., 2016). However, mitosis of circulating haemocytes has been suggested as the primary source of haemocyte replenishment during pathogenic infection in both mosquitoes and locusts (Duressa et al., 2015; King and Hillyer, 2013).

Based on our observations in *O. chinensis* and other reports, the study of the morphology and histochemistry of haemocytes is essential to the understanding of their function. Plasmatocytes and granulocytes are the primary phagocytic cells of *O. chinensis* based on their abundance, morphology, histochemistry and phagocytic responses. Our histochemical stains indicate that the haemocytes of *O. chinensis* are capable of digesting foreign materials, detoxicating haemolymph and regulating metabolism. The importance of non-phagocytic haemocytes found in this study to the cellular response of *O. chinensis* is questionable due to their low abundance and unknown histochemical properties. Based on our results, we believe light microscopy observation with stained preparations is still convincing in examining the morphology, histology and function of insect haemocytes. The results of this study may contribute to the improvement and standardisation of the staining techniques used in studies of insect haemocytes. Morphological features alone are not sufficient to confirm the classification of insect haemocytes, and combinations of techniques are needed to confirm haemocyte type and function.

## MATERIALS AND METHODS

### Species

*O. chinensis* adult males were collected from Jinyuan District, Taiyuan, Shanxi Province, China (latitude: 37°42′24.51″; longitude: 112°26′33.05″; altitude: 779.72 m). The collected samples were taken to the laboratory alive, kept in a well-ventilated room and fed with rice. They were maintained for a week before blood sampling and treatment.

### Microscopy

Haemolymph was extracted from the prothoracic leg bases of each grasshopper*.* The ethanol-sterilised leg base skin was pierced and blood was taken with a pipette (MicroPette Plus, Scilogex, Rocky Hill, CT, USA). The blood smears were prepared for light (Olympus BX-51, Tokyo, Japan) and scanning electron microscopy (Hitachi S-570). For light microscopy, smears were prepared conventionally and stained with Wright-Giemsa dye and four histochemical procedures: ACP reaction, PAS reaction, ORO staining and CAE reaction. All dyes were purchased from Baso Diagnostics Inc. (Guangdong, China). After staining, all smears were washed with water, air-dried and mounted with neutral balsam.

For the Wright-Giemsa stain, smears were stained with Wright-Giemsa staining solution for 10–20 min at room temperature. For ACP, smears were fixed in an acetone-methanol buffer for 30 s at 4°C and stained with Acid Phosphatase staining solution for 47 min at room temperature. Washed and air-dried smears were counterstained with Hematoxylin staining solution for 5 min. The positive-reacted particles were purplish red. For PAS, smears were fixed with 95% ethanol for 10 min, and immersed in Periodic Acid solution (10 g/l) for 20 min and Schiff solution for 30 min at room temperature. Smears were then washed with water, air-dried and counterstained with Hematoxylin for 5 min. The presence of red granules or masses in the cytoplasm confirmed a positive reaction. For ORO, smears were fixed with formaldehyde vapour for 10 min and air-dried for 15 min, followed by a 15 min immersion in the Oil Red O dye, then rinsed with 60% isopropyl alcohol and then water. A counterstain with Hematoxylin for 2 min and Disodium Hydrogen Phosphate solution was performed for 1 min. Neutral lipids were stained red, phospholipids were stained pink and nuclei were stained blue. For CAE, the smears were fixed in the Acetone-Methanol buffer for 30 s at 4°C. Washed smears were then stained in the substrate solution for 30 min and counterstained with Hematoxylin staining solution for 10 min at room temperature. Positively reacted cells contained red particles.

For scanning electron microscopy, smears were fixed in 3% glutaraldehyde, washed with 0.1 M phosphate (pH 7.4) and post-fixed with 1% osmium tetroxide. Fixed smears were then dehydrated with ethanol in a graded series (20%, 40%, 60%, 75%, 90%, 95% and 100%) followed by a graded series of tertiary butanol. Dehydrated smears were freeze-dried with an FD-1A-50 freeze dryer (Beijing, China) and coated with gold using an SBC-12 ion sputter coater (Shanghai, China).

For transmission electron microscopy (JEM-1400), extruded blood was fixed in tubes with 5% glutaraldehyde. The tubes were centrifuged at 3000 rpm for 5 min, and the supernatant was discarded. Haemocyte pellets were post-fixed with 1% osmium tetroxide, and dehydrated in a graded ethanol series followed by acetone, before being embedded in Epon812 resin. Sections were cut with an ultramicrotome (Leica RM2255) and stained with uranyl acetate followed by lead citrate.

### Bacterial infection experiment

*E. coli*, *B. subtilis* and *S. aureus* bacteria were purchased from China General Microbiological Culture Collection Centre (Beijing, China). Thirty adult grasshoppers were separated into three groups and injected, respectively, with 10 μl of *E. coli*, *B. subtilis* or *S. aureus* in a (1×10^5^ ml^−1^) suspension of phosphate buffered saline (pH 7.4). Bacteria were injected using a microsyringe between the second and third segments of the abdomen, following ethanol (75%) sterilisation. All grasshoppers were alive at the end of the experiment. Their thoraxes were disinfected with ethanol and pierced with a needle to extract haemolymph at 4, 8, 12, 24 and 48 h after treatment. A gentle squeeze of the thorax was applied to promote bleeding, and a pipette was used to collect blood. Slides were prepared and stained with Wright-Giemsa as described previously.

### Haemocyte count and statistical methods

Different haemocyte types were counted on the Wright-Giemsa-stained slides, and their relevant percentages were calculated. Ten untreated grasshoppers were selected, and at least 400 cells were counted from each. The number of phagocytic haemocytes from the bacteria-treated grasshoppers was counted at 4, 8, 12, 24 and 48 h after injection. Statistica 10 data analysis software (StatSoft Inc., 2011) was used to run statistical tests and generate graphs. Data obtained from the differential counts were analysed using ANOVA and Tukey's Honestly Significant Difference test (α=0.05).
